# An outbreak of cardiovascular syndromes requiring urgent medical treatment and its association with environmental factors: an ecological study

**DOI:** 10.1186/1476-069X-6-37

**Published:** 2007-11-25

**Authors:** Robin M Turner, David J Muscatello, Wei Zheng, Alan Willmore, Glenn Arendts

**Affiliations:** 1 New South Wales Department of Health, 73 Miller St, North Sydney NSW 2060 Australia; 2 St George Hospital and Community Health Service, South Eastern Sydney and Illawarra Area Health Service, Gray Street Kogarah NSW 2217 Australia

## Abstract

**Background:**

In April 2005, syndromic surveillance based on statistical control chart methods in Sydney, Australia, signalled increasing incidence of urgent emergency department visits for cardiovascular and chest pain syndromes compared to the preceding twelve months. This paper aimed to determine whether environmental factors could have been responsible for this 'outbreak'.

**Methods:**

The outcome studied was daily counts of emergency department visits for cardiovascular or chest pain syndromes that were considered immediately or imminently life threatening on arrival at hospital. The outbreak had a mean daily count of 5.7 visits sustained for eight weeks, compared with 4.0 in the same months in previous years. Poisson regression was used to systematically assess the emergency department visits in relation to available daily weather and pollution variables by first finding the best model that explained short-term variation in the outcome over the period 25 January 2002 to 31 May 2005, and then assessing interactions of all available variables with the 'outbreak' period, April-May 2005. Rate ratios were estimated for an interquartile increase in each variable meaning that the ratio measures the relative increase (or decrease) in the emergency department visits for an interquartile increase in the weather or pollution variable. The rate ratios for the outbreak period measure the relative increase (or decrease) in the emergency department visits for an interquartile increase in the weather or pollution variable during the outbreak period only.

**Results:**

The best fitting model over the whole study period included minimum temperature with a rate ratio (RR) of 0.86 (95% confidence interval (CI), 0.77–0.96), maximum relative humidity of 1.09 (95% CI 1.05–1.14) and minimum daily particulate matter less than 10 microns (PM_10_) of 1.05 (95% CI, 1.01–1.09). During the outbreak period, maximum temperature (RR 1.27, 95% CI 1.03–1.57), solar radiation (RR 1.44, 95% CI, 1.00–2.07) and ozone (RR 1.13, 95% CI 1.01–1.26) were associated with the outcome.

**Conclusion:**

The increase may have been associated with photochemical pollution. Syndromic surveillance can identify outbreaks of non-communicable diseases associated with environmental factors.

## Background

Syndromic surveillance is used for early warning of emerging health problems in populations, however there is sparse documentation on the application of syndromic surveillance for non-communicable disease outbreaks and, particularly, response options for syndromic surveillance signals. The New South Wales (NSW) Department of Health, Australia, operates near real-time syndromic surveillance using automatic capture and analysis of electronic emergency department information system data [1].

In April 2005, the surveillance system signalled an increase in the incidence of admissions to the critical care wards of St George Hospital in Sydney. Subsequent analysis found that cardiovascular syndromes comprised the majority of the increased critical care admissions, and that hospital admission practices could not explain this increase.

There is mounting evidence for short-term adverse effects of environmental factors, including air pollution, on cardiovascular morbidity and mortality, both within Australia and internationally [2,3]. In particular, increased levels of air pollution are associated with increased emergency department attendances for cardiovascular diseases [4,5].

Given the known association between environmental factors and cardiovascular disease outcomes, and the unusual extended period of dry, mild, stable weather conditions arising from drought conditions at the time [6], we hypothesised an environmental cause of the observed increase in more urgent emergency department visits for cardiovascular or chest pain syndromes in the region surrounding the hospital. This report describes the initial assessment of the surveillance signal and the results of a study designed to assess the role of environmental exposures in the observed phenomenon.

## Methods

The surveillance system groups patient visits into mutually exclusive syndromes by using the provisional emergency department diagnosis coded in either International Classification of Diseases (ICD) version 9 or version 10. The diagnosis codes included in the cardiovascular and chest pain syndromes were: ICD-9: 390–459, 786.5, V12.5, and 785; and ICD-10: G45, I00–I99, R07.1–R07.4, Z86.7 and R00–R03. Another set of 'severity syndromes' relate to the urgency and severity of visits and are not tied to diagnosis groups. These severity syndromes include an 'admission to critical care ward' syndrome using the patient's disposition on departure from the emergency department.

Once the surveillance signal occurred, we allocated the provisional diagnoses of visits admitted to critical care into our diagnosis-based syndromes and assessed how their relative contribution to recent activity had changed. This implicated cardiovascular disease and chest pain syndromes in the increase. Consultation with clinical personnel (emergency department physicians, critical care physicians and cardiac physicians) at the hospital did not reveal an explanation for the increase based on clinical practice.

The trend continued and no explanation had been found by the end of May 2005. To exclude confounding by changes in hospital practice after entry into the emergency department, we examined trends in cardiovascular and chest pain syndromes by triage category. On arrival at the emergency department, each patient is assigned a triage code from most urgent (1) to least urgent (5) [7] according to the triage nurse's assessment of the urgency of medical treatment required. Cardiovascular conditions that typically would be assigned code 1 or 2 include cardiac arrest, acute pulmonary oedema, haemodynamically unstable arrhythmia or chest pain that is likely cardiac in nature.

Using electronic databases and medical record review at the hospital, we compared clinical characteristics of patients for the period 1 April to 31 May 2005 with characteristics of patients in the same period in the previous three years.

We now describe the design of an environmental study to evaluate environmental factors that may have been associated with this phenomenon.

The 'outbreak' period was defined as 1 April to 31 May 2005; during the Australian autumn (fall). To allow comparison with past environmental exposures, we chose a prior comparison period that allowed us to use the maximum amount of environmental data available from the pollution sites used in the study: 25 January 2002 to 31 March 2005.

**Figure 1:**
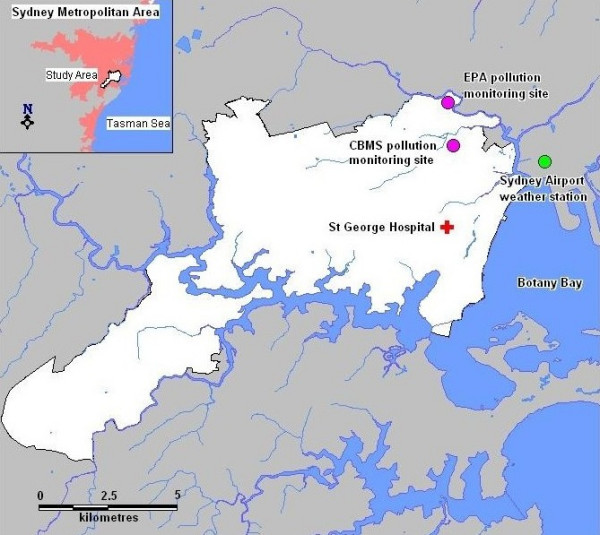
**Study area**. The study area shown in white with the location of the pollution and weather monitoring stations and St George Hospital.

St George Hospital is a major metropolitan public hospital, receiving approximately 48,000 emergency department visits per year. It is located in the densely populated, low-lying eastern coastal part of the Sydney basin, close to Sydney Airport. Although the increase in visits was identified by surveillance of this hospital, an environmental exposure would be geographically based and not hospital-based. We therefore defined the study area to be the boundaries of all postal codes from which the hospital drew at least one per cent of its visits during the outbreak period (Figure 1), and included emergency department visits for all persons residing in the study area, regardless of hospital attended. The study area occupies 124 square kilometres, and had a population in 2001 of 299,449. This represents an average population density of over 2,400 (range 653–22,353) persons per square kilometre, which places it among the highest population densities in NSW.

Triage category is assigned using a common scale at all emergency departments in NSW, and admission practices can vary between hospitals. We therefore made a prior decision to use triage category rather than critical care admissions as the study outcome. Included in the study were daily counts of visits to any emergency department where the patient's address of residence was within the study area, the triage category assigned on arrival was either immediately or imminently life-threatening or of time-critical treatment acuity (categories 1 or 2), and the provisional emergency department diagnosis met our definition of cardiovascular or chest pain syndromes. Because at the time the surveillance system had incomplete hospital coverage, we obtained visit data from the NSW Emergency Department Data Collection (HOIST). The HOIST system refers to a data access, analysis and reporting facility established and operated by Centre for Epidemiology and Research, Public Health Division, NSW Health Department. This emergency department data collection has complete coverage of Sydney public hospital emergency departments and most larger rural emergency departments [8] but the information is reported more slowly to the Department of Health than in the surveillance system. At the time the analysis was conducted, the Emergency Department Data Collection had complete data up to and including 31 May 2005.

Weather data was obtained from the Australian Bureau of Meteorology [9] for the Sydney Airport Monitoring Station, which is the closest Bureau of Meteorology monitoring station to the study area. Hourly data was available for the following variables: air temperature, dew point temperature, relative humidity, pressure, wind speed and direction, and precipitation. Solar radiation was available from one of the pollution monitoring sites as described below.

Two sources of pollution data were obtained. The NSW Department of the Environment and Conservation (EPA) [10] has pollution monitoring sites throughout the Sydney area. The only monitoring site within the study area is at Earlwood (Figure 1). Data obtained from the Earlwood monitoring site over the study period contains measurements of PM_10_, particulate matter less than 2.5 microns (PM_2.5_), particulate matter as determined by the integrating nephelometer method (NEPH), nitrogen dioxide (NO_2_) and ozone.

The NSW Roads and Traffic Authority [11] monitors pollution from vehicles near a motorway on the northern fringe of the study area. Four monitoring sites are located within the study area. We chose the community based monitoring station (CBMS) site for this study, as it is located within a residential part of the study region and is closest to the St George Hospital (Figure 1). Measurements were obtained from the CBMS for the pollutants PM_10_, carbon monoxide (CO), NO_2 _and nitrous oxide (NO). Solar radiation was also available from CBMS. Even though the CBMS site was slightly closer to the hospital than the Earlwood site we included variables measured at Earlwood because additional variables were available from Earlwood (PM_2.5_, NEPH and ozone).

Weather and pollution data were recorded at various frequencies from the weather station and the two pollution monitoring sites; five minutes, thirty minutes and hourly. Daily mean, maximum and minimum values were calculated from the hourly value or the hourly average of the five and thirty minute recorded values. Missing weather or pollution data meant 7% of daily observations were excluded from the final analysis.

Because we had no prior hypothesis about the potential cause of the increase in visits, model building using Poisson regression was used to find the best fitting model that predicted the outcome variable over the entire study period. This allowed us to determine if variables that were associated with short-term changes in the outcome over a longer period also predicted the change in the outcome during the outbreak period.

First, a baseline model containing potential confounders identified by other studies was developed. These included day of week, sine and cosine terms accounting for seasonal trend, linear and quadratic trend, school holidays, public holidays and the influenza season. The influenza season variable used daily counts of emergency department visits by residents of the study area that were assigned a provisional diagnosis of influenza (ICD-9 code 487; ICD-10 code J10–J11). In NSW, counts of these diagnoses follow influenza epidemics quite closely [12]. All models were fitted using SAS System version 8.02 [13].

Second, we univariately screened the large number of weather variables, each with lags from 0 to 7 days, to determine those that revealed a significant association with the outcome after adding them to the baseline model. Significant variables from the univariate analysis were then combined in a stepwise backwards regression to identify the weather variables that provided the best model fit according to the Akaike Information Criteria (AIC) [14]. Interactions between those weather variables and the confounders included in the first stage of the modelling were then investigated. Any interactions that improved model fit were retained. This became the 'best weather model'.

Third, a similar process was undertaken to screen the pollution variables and arrive at the 'best pollution model'. Each pollution variable was included univariately with a 0 to 7 day lag in the best weather model. Any significant pollution variables (at the 5% level) were then combined in a stepwise backwards regression to arrive at the best pollution model, based on AIC. A further check was done for interactions between the pollution variables and the confounders.

Finally, as the focus of the study was to explain what happened during the outbreak period, we defined an indicator variable equal to zero during the non-outbreak period and one during the outbreak period. We then univariately included each of the weather and pollution variables lagged from zero to seven days into the best pollution model with an interaction of that variable with the outbreak period indicator variable. This allowed us to assess which variables were associated with the outcome during the outbreak period.

Given the limited power offered by the relatively short outbreak period, we did not attempt to assess multiple interactions with the outbreak period.

The rate ratios from the Poisson regression measure the multiplicative increase (or decrease) for an interquartile increase (an increase from the 25^th ^to the 75^th ^percentile) in the weather and pollution variables. The 25th and 75th percentiles were calculated from the whole analysis period.

## Results

### Initial surveillance signal assessment

**Figure 2:**
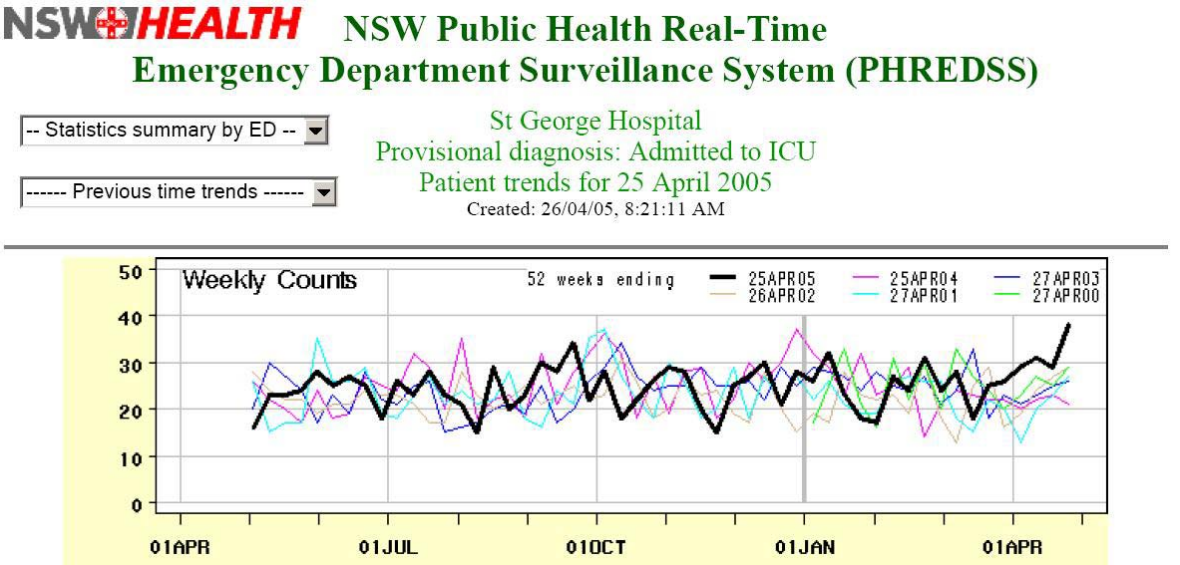
**Initial emergency department surveillance graph**. Surveillance system graph comparing weekly counts of critical care admissions to St George hospital with the same weeks in previous years when first signalled.

**Figure 3:**
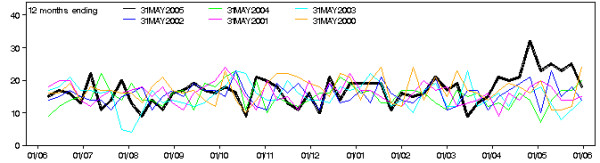
**Emergency department surveillance graph entire study period**. Surveillance system graph comparing weekly counts of critical care admissions to St George hospital with the same weeks in previous years up to the end of the outbreak.

On 26 April 2005, the surveillance system signalled an increase in visits admitted to the critical care ward to St George Hospital (Figure 2) based on a cumulative sum statistical control chart technique [1]. The standard surveillance report graphs weekly activity with previous years and showed a clear short-term increase against both recent and seasonal background trends. Assigning diagnosis-based syndromes to the critical care admissions revealed that the contribution of cardiovascular and chest pain syndromes had increased during that month. Figure 3 shows the entire outbreak period on top of the previous years data. There is a clear sustained increase in the weekly counts.

**Figure 4:**
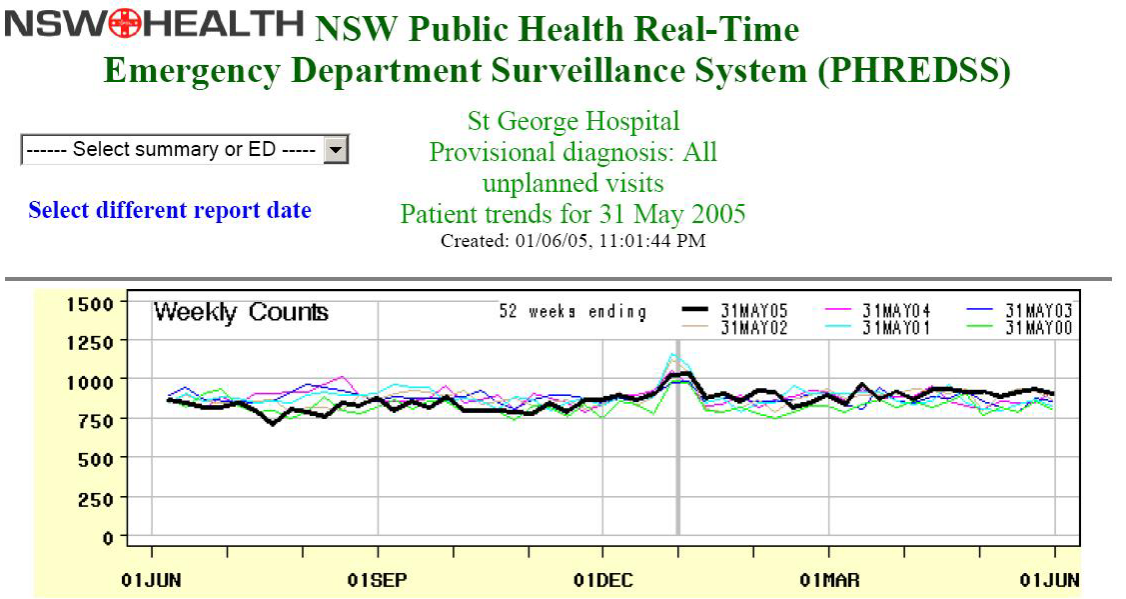
**Emergency department surveillance graph all visits**. All unplanned visits to St George emergency department for any condition.

Figure 4 shows all unplanned emergency department visits during the study period. Whilst there was a substantial increase in cardiovascular and chest pain symptoms requiring urgent treatment there was only a small increase in the overall number of emergency department visits relative to previous years. This may have been at least partly explained by the increase in cardiovascular and chest pain syndromes.

Counts of patients with cardiovascular or chest pain syndromes that were assigned either of the two most urgent triage categories (1 and 2) increased coincidentally with the critical care ward admissions, leading us to conclude that the change in critical care ward admissions was not due to clinical practices once inside the emergency department. We confirmed with the hospital that there we no system or coding changes that could explain the increase. The increase was also seen in patients arriving by their own transport, so a change in ambulance deployment could not explain the increase.

Potential confounding by hospital practice once inside the emergency department led us to restrict subsequent analyses to visits with a presumptive cardiovascular diagnosis (including chest pain) and triage category 1 and 2 combined. Table 1 compares the clinical characteristics of these patients in the outbreak period with the same period in the previous three years. Patients in the outbreak period were more likely to be female (46% female in the outbreak compared to 37% in the non-outbreak period, p = 0.03) and less likely to be discharged from the emergency department but there were otherwise no statistically significant differences between the populations in terms of hospital length of stay, final discharge diagnosis from hospital or procedures done as an inpatient. The number of patients seen during the two-month outbreak was 247 compared with an average of 146.0 for the same two months in the previous three years, an increase of 69%. This suggests the overall numbers of patients in the outbreak period increased but with little difference in the distribution of disease seen. Population increases would be unlikely to explain the outbreak given the short-term increase.

**Table 1:** Clinical characteristics. Clinical characteristics of St George emergency department patients with triage code 1 or 2 and provisional ED diagnosis of chest pain or cardiovascular disease.

		Comparison period (April & May 2002–2004)		Outbreak period (April & May 2005)	
		
		Average count per year	%	Count	%
Age in years	(median; IQR)	71; 56–81		70; 55–82	

Age group	0–34 years	7.0	5%	9	4%
	35–64 years	51.3	35%	87	35%
	65 years and over	87.7	60%	151	61%

Male		92.0	63 %	134	54%

Disposition from ED	Discharged from ED	26.3	18%	20	8%
	Admitted to ward	55.3	38%	89	36%
	Admitted to critical care	51.7	35%	128	52%
	Died in ED	11.3	8%	7	3%
	Transferred to other facility	1.3	1%	3	1%

Hospital length of stay in days	(median; IQR)	4; 2–8		4; 1–8	

Major procedures in hospital	Coronary angiography +/- stent insertion	27.0	25%	48	22%
	Coronary artery bypass grafting	6.7	6%	10	5%
	Cardioversion	1.0	1%	3	1%
	Insertion of pacemaker	2.0	2%	4	2%

Final disposition from hospital	Home	92.3	86%	179	82%
	Transferred to other facility	8.3	8%	26	12%
	Died	6.3	6%	12	6%

Final discharge diagnosis	Acute myocardial infarction	29.3	27%	51	24%
	Angina	14.3	13%	29	13%
	Non cardiac chest pain	11.7	11%	40	18%
	Arrhythmia	14.7	14%	26	12%
	Cardiac failure	10.3	10%	15	7%
	Other	26.7	25%	56	26%

Total		146.0	100%	247	100%

Length of stay, procedures and final disposition and diagnosis only available for patients admitted to an inpatient ward

### Environmental study results

The characteristics of the sample of emergency department visits selected geographically were not substantially different from the subset of St George Hospital visits in terms of age, sex and disposition. In the geographic sample, the mean daily count during the outbreak was 5.7 compared with 4.0 during the entire study period prior.

Table 2 shows the distribution of the daily measurements of the weather and pollution variables during the study period overall, during the outbreak period, and the average for that period in earlier years. Median PM_10 _and PM_2.5 _levels were higher during the outbreak period than both the same period in earlier years and compared with the whole study period. Median nephelometry readings during the outbreak period were not unusual for the time of year. Median CO readings were low for the time of year. Median NO_2 _and NO readings were slightly high for the time of year and higher than the study period overall. NO readings in particular were dramatically higher during the outbreak generally compared with the study period overall, with the median of the mean daily NO reading being between 24.5 and 27.0 parts per hundred million compared with 11.3 for the study period overall. Median ozone readings were slightly high for the time of year but were lower than the study period overall.

**Table 2:** Descriptive statistics. Descriptive statistics (percentiles) of weather and pollution variables during the study period 25 Jan 2002 and 31 May 2005.

		Comparison period (April & May 2002–2004)				Outbreak period (April & May 2005)				Total period			
		
	Daily	N	25th	50th	75th	N	25th	50th	75th	N	25th	50th	75th
	
PM10 CBMS^a ^(ug/m^3^)^b^	Min	183	3.2	5.7	9.3	55	4.3	8.6	13.3	1183	3.0	6.5	10.8
PM10 Earlwood^c ^(ug/m^3^)	Min	183	4.0	7.2	11.7	61	6.8	9.5	12.5	1191	4.0	7.7	12.2
PM2.5 Earlwood (ug/m^3^)	Mean	183	7.5	10.0	13.3	61	8.6	10.6	13.2	1216	7.6	10.0	13.4
NEPH^d ^Earlwood	Mean	176	0.2	0.2	0.4	61	0.2	0.2	0.3	1209	0.1	0.2	0.3
CO CBMS (ppm)^e^	Mean	181	0.1	0.3	0.5	59	0.1	0.2	0.4	1187	0.1	0.1	0.3
NO2 CBMS (ug/m^3^)	Mean	183	24.9	32.1	40.5	57	25.2	33.6	42.2	1183	17.5	26.7	37.8
NO2 Earlwood (pphm)^f^	Mean	182	1.2	1.5	1.8	61	1.1	1.5	1.8	1189	0.8	1.3	1.7
NO CBMS (ug/m^3^)	Mean	183	10.3	24.5	46.9	57	12.9	27.0	57.8	1183	3.4	11.3	29.8
Ozone Earlwood (pphm)	Max	177	2.2	2.6	3.0	61	2.5	2.7	3.1	1199	2.3	2.8	3.3
Relative Humidity Airport^g ^(%)	Max	183	82.0	90.0	95.0	61	85.5	89.0	91.0	1223	78.5	87.5	93.0
Air Temperature	Min	183	12.5	14.3	16.4	61	12.9	15.0	17.2	1194	11.2	15.3	18.6
Airport (°C)	Max	183	19.1	21.0	23.0	61	20.0	22.3	24.4	1223	19.0	22.1	25.3
Solar Radiation CBMS (w/m^2^)	Mean	183	108.6	143.6	166.8	61	110.6	148.8	166.6	1223	127.5	174.8	258.6
Precipitation Airport (mm)	Max	183	0.0	0.1	5.2	61	0.0	0.0	0.4	1223	0.0	0.0	2.0
Station Pressure Airport (hPa)	Mean	183	1015.4	1020.0	1023.6	61	1018.6	1021.9	1024.3	1223	1012.2	1016.9	1021.3
Wind Speed Airport (km/h)	Mean	183	13.3	15.9	21.8	61	12.9	16.7	19.7	1223	15.0	18.8	23.7

^a ^A community based monitoring station of the Road and Traffic Authority.

^b ^ug/m^3 ^= micrograms per cubic meter, i.e. mass of pollutant per volume of air.

^c ^A monitoring station of the Environmental Protection Agency at Earlwood.

^d ^Coefficient of light scattering by fine particles per 10 km.

^e ^ppm = parts per million by volume, i.e. parts of million parts of air.

^f ^pphm = parts per hundred million by volume, i.e. parts of pollutant per hundred million parts of air.

^g ^A weather monitoring station at the Sydney Kingsford-Smith Airport.

The median of the daily maximum relative humidity readings during the outbreak period was slightly low for the time of year but was higher than for the study period overall. Minimum and, particularly, maximum daily temperatures were high for the time of year. The bottom 25th percentile and the median of the daily mean solar radiation were high for the time of year, but the upper 75th percentile was not unusual. This suggests reduced cloud cover during the outbreak period, consistent with the drought conditions at the time. Mean daily air pressure readings were higher during the outbreak period than both the same period in earlier years and the study period overall and showed less variation. The median of the mean daily windspeed during the outbreak period was high for the time of year, but lower than the study period overall. The 25th and 75th percentiles of mean windspeed were lower than the same period in previous years and the study period overall. The relatively low standard deviations of maximum relative humidity, air   pressure and windspeed indicate lower variability in these variables during the outbreak period for the time of year (figures not shown). Maximum daily precipitation was dramatically lower during the outbreak period than the study period overall and the same period in previous years, confirming the drought conditions (Table 2).

The best-fitting baseline weather model, after univariate inclusion of each weather variable, included daily maximum relative humidity (rate ratio (RR) 1.08, 95% CI 1.04–1.13) and daily minimum air temperature (RR 0.87, 95% CI 0.78–0.97) each at a lag of three days, and unlagged daily mean solar radiation (RR 1.07, 95% CI 1.01–1.13). Solar radiation showed a significant interaction with day of the week. Although its interaction with public holidays was not statistically significant, the interaction improved model fit so it was retained in the baseline weather model.

After univariately including each lagged value of the pollution variables into the baseline weather model, significant positive associations with the outcome were found for mean PM_2.5 _(RR 1.03, 95% CI 1.00–1.07) and maximum nephelometry at Earlwood lagged one day (RR 1.02, 95% CI 1.00–1.04), maximum PM_2.5 _at Earlwood lagged one (RR 1.03, 95% CI 1.00–1.05,) and two (RR 1.03, 95% CI 1.00–1.05) days, minimum CO at CBMS lagged one day (RR 1.01 95% CI 1.00–1.01), and minimum PM_10 _lagged three days at CBMS (RR 1.05, 95% CI 1.01–1.10). Minimum PM_10 _at CBMS lagged three days provided the best fit and was retained for the best single pollutant model.

Inclusion of the outbreak period indicator variable, which has a value of one during the outbreak and zero in the non-outbreak period, confirmed a statistically significant increase of 24% (95% CI 4%-46%) in the mean daily count of the outcome during April-May 2005, after adjusting for all other variables in the model at that stage.

The results are shown in Table 3. The first column shows the different period interaction models with each variable that showed significant interactions with the outbreak period down the rows, the first row is the best single pollutant model with no interaction. The next four columns show the rate ratios for the variables from the best single pollutant model that included minimum air temperature (lag 3 days), maximum relative humidity (lag 3 days), mean solar radiation (lag 0 days) and minimum PM10 (lag 3 days). The next two columns give the rate ratios for the period and period interaction variables for the particular model of interest. The final two columns show the rate ratio in the outbreak period, indicating the relative change in the outcome for an interquartile increase in the variable during the outbreak period, and the rate ratio in the non-outbreak period which measures the relative change in the emergency department visits for an interquartile increase in the variable during the non-outbreak period only. For example mean solar radiation (no lag) had a significant interaction with the period variable (rate ratio = 1.48, 95% CI (1.04 – 2.1)). Mean solar radiation showed little relationship to the emergency department visits during the non-outbreak period (rate ratio = 0.97, 95% CI (0.86 – 1.10)) but during the outbreak period an interquartile increase in mean solar radiation was associated with a 44% (rate ratio = 1.44) increase in the emergency department visits with 95% CI (1.00 – 2.07). The variables ozone, air temperature (both minimum and maximum) and solar radiation all show a different, more harmful relationship with the outcome during the outbreak period. The confidence intervals are much wider during the outbreak period because of the much smaller amount of data being used for the estimation.

**Table 3:** Model Results. Rate ratios (95% confidence intervals) from the models assessing interactions of the weather and pollution variables with period (outbreak vs non-outbreak) within the best single pollutant model.

	Best single pollutant model variables				Period by variable interaction terms			
Model	Minimum air temperature (lag 3 days)	Maximum relative humidity (lag 3 days)	Mean solar radiation (no lag)	Minimum PM10 (lag 3 days)	Period	Interaction variable	Rate ratio in outbreak	Rate ratio in non-outbreak

Best single pollutant model	0.86 (0.77 – 0.96)	1.09 (1.05 – 1.14)	0.81 (0.59 – 1.11)	1.05 (1.01 – 1.09)	NA	NA	NA	NA
Period by mean solar radiation (no lag)	0.86 (0.77 – 0.96)	1.09 (1.04 – 1.14)	0.97 (0.86–1.1)	1.05 (1.01 – 1.09)	0.80 (0.51 – 1.23)	1.48 (1.04 – 2.1)	1.44 (1.00 – 2.07)	0.97 (0.86–1.10)
Period by max ozone (lag 1 day)	0.87 (0.78 – 1.97)	1.09 (1.04 – 1.14)	1.01 (0.89 – 1.14)	1.05 (1.01 – 1.09)	0.86 (0.58 – 1.27)	1.13 (1.01 – 1.27)	1.13 (1.01 – 1.26)	1.00 (0.97–1.03)
Period by Max ozone (lag 5 days)	0.85 (0.76 – 0.95)	1.09 (1.05 – 1.14)	0.98 (0.87 – 1.11)	1.05 (1.01 – 1.09)	0.87 (0.60 – 1.28)	1.12 (1.00 – 1.25)	1.11 (0.99 – 1.24)	0.99 (0.96–1.01)
Period by max air temp (no lag)	0.86 (0.77 – 0.96)	1.09 (1.04 – 1.13)	0.99 (0.88 – 1.11)	1.05 (1.01 – 1.09)	0.46 (0.21 – 1.01)	1.32 (1.07 – 1.64)	1.27 (1.03 – 1.57)	0.96 (0.91–1.01)
Period by min air temp (no lag)	0.86 (0.77 – 0.96)	1.09 (1.04 – 1.13)	0.98 (0.87 – 1.11)	1.05 (1.01 – 1.09)	0.64 (0.34 – 1.20)	1.41 (1.03 – 1.92)	1.35 (0.99 – 1.85)	0.96 (0.86–1.08)
Period by min air temp (lag 4 days)	0.83 (0.73 – 0.94)	1.09 (1.05 – 1.14)	0.98 (0.87 – 1.11)	1.05 (1.01 – 1.09)	0.42 (0.20 – 0.90)	1.71 (1.18 – 2.46)	1.77 (1.22 – 2.58)	1.04 (0.92–1.18)
Period by mean solar radiation (lag 5 days)	0.86 (0.77 – 0.96)	1.09 (1.04 – 1.13)	0.98 (0.87 – 1.11)	1.05 (1.01 – 1.08)	0.84 (0.55 – 1.27)	1.41 (1.01 – 1.96)	1.38 (0.99 – 1.92)	0.98 (0.93–1.04)

NA = Not Applicable

The rate ratios for the variables from the best weather and pollution model (shown down the first four columns) vary little when the different period by variable interaction terms are included. The association of minimum PM_10 _with the outcome did not change over the outbreak period. The period variable is not significant when the interactions for solar radiation, ozone and air temperature are included (separately) indicating that their interaction with the period variable explains much of the actual increase during the period.

The maximum correlation between variables in any one model was 0.4 for maximum ozone and mean solar radiation; this was not significantly different from zero. This indicates collinearity was not a concern in the modelling process.

## Discussion

In this outbreak of cardiovascular syndromes identified by syndromic surveillance, we found that while humidity and PM_10 _were positively associated with daily counts of more urgent emergency department visits for cardiovascular and chest pain syndromes over the entire study period, temperature, solar radiation and ozone were all positively associated with the outcome during the outbreak period. PM_10 _and humidity did not have a different association during the outbreak period of April-May 2005.

In our study, initial analysis showed that the outbreak was marked by an increase in the overall number of patients with cardiac diagnoses rather than any change in the severity or type of illness seen. This would point to environmental factors influencing the frequency of presentations among susceptible individuals rather than among the broader, low-risk population.

Both solar radiation and temperature are important catalysts in photochemical smog reactions that produce ozone and other oxidants [15-17]. Temperature and solar radiation were unseasonally high during the outbreak period. The addition of drought and high atmospheric pressure provided favourable conditions for atmospheric stagnation and ozone build-up [18]. The reduced variability in air pressure during the outbreak period provides further support to a stagnation hypothesis.

Evidence for the effect of ozone on cardiovascular outcomes is emerging [19-22], including, more specifically, cardiac arrhythmias [23]. Ozone has been associated with cardiovascular emergency department visits, but at concentrations higher than those recorded in our study [24]. Yet ozone levels were only slightly unseasonally high during the outbreak period and were lower than at other times during the study period. Further, temperature and solar radiation exposure would be lower during the outbreak period than in summer. This points to some unmeasured factor. Ozone levels measured at Earlwood may not have been representative of personal ozone exposures; the monitoring site was on the fringe of the study area and personal ozone exposures can vary substantially from ambient measures [15]. Unmeasured seasonal factors may have been important. Seasonal differences in cardiovascular outcomes have been reported in low pollution environments [4,25]. Furthermore, measurements of sulphur dioxide and many other pollutants were unavailable for our study. Sulphur dioxide has been associated with adverse cardiovascular outcomes [24-26], including the Sydney setting [4]. Additionally, there is now evidence that solar radiation stimulates the degradation of certain volatile organic compounds, such as isoprene and 1,3-butadiene, into chemical by-products that are both toxic and pro-inflammatory to lung cells [27]. Biological mechanisms linking lung inflammation to cardiovascular effects have been proposed [28]. Isoprene and 1,3-butadiene arise from vehicular emissions and other human and natural sources [29,30]. Associations between volatile organic compounds and ischaemic heart disease visits have been reported [5]. Ultrafine particles (diameter less than 100 nanometres), also unmeasured in our study, have also been implicated in inflammatory and toxic effects potentially dangerous to the cardiovascular system [31,32]. Strong seasonal differences in the generation of ultrafine particles from traffic have been reported, and solar radiation and temperature were mediators in the generation of these particles, possibly through photochemical reactions [33].

While PM_10 _was implicated in our study for the entire period, we did not find evidence that it was directly responsible for the observed increase during the outbreak period. One possible reason could be that ambient monitoring did not represent personal exposures during the outbreak period [34]. The lack of strong correlation between minimum PM_10 _values at the CBMS monitoring site with those at Earlwood (r = 0.60) and the stronger correlation between maximum values at the two sites (r = 0.75) suggests minimum daily values may be more sensitive to local conditions than maxima.

There is substantial coherent epidemiological and biological evidence for the effect of PM_10 _on cardiovascular outcomes [28], although we were unable to find literature relating to the association of *minimum *PM_10 _measurements on health outcomes. We speculate that high *minimum *PM_10 _values during the day could represent prolonged low-level exposures, for example, due to lack of clearing caused by atmospheric stagnation or sustained traffic volume.

The nature of diagnosis coding in emergency departments is not usually reported in air pollution studies that use this outcome. Only [24] reported the use of manually reviewed medical records in assigning the diagnosis of emergency department visits. Our study, like other studies that use routinely recorded information from patient information management systems, used the provisional diagnosis at the time of the ED visit, not the final diagnosis on discharge from the hospital. Many provisional diagnoses in our emergency department systems are symptoms, rather than confirmed diagnoses and we therefore included the chest pain diagnosis in our analysis. This decision was supported by the initial analysis of critical care admissions first observed.

Our inclusion of the chest pain diagnosis with cardiovascular diagnoses and our restriction on more urgent visits makes it difficult to compare our findings with other studies. Nevertheless, PM_10 _has been found to be associated with cardiovascular visits in Sydney, particularly in the cooler part of the year [4]. Other pollutants have also been associated with cardiovascular visits: PM_2.5 _and NO_2 _[4,5]; CO [4,5,35]; ozone [24]; sulphur dioxide [4,24]; particulate carbon and oxygenated hydrocarbons (volatile organic compounds) [5]. We had measurements for PM_2.5_, CO, NO_2 _and ozone, but only ozone was implicated in our outcome and only during the outbreak period.

The only study we could find that assessed an outcome of more urgent cardiovascular disease morbidity [36], found that PM_10 _and NO_2 _in Tokyo, where the reported levels were markedly higher than those in our study, were associated with emergency transports of patients for angina, cardiac insufficiency and myocardial infarction. Only the summer months were studied, however.

The pollution and weather data used in our study could only be obtained from sites on the fringes of the study region. For pollution data, this is less than ideal because exposures may have varied within the study region as discussed earlier. This would not, however, be a concern for the weather and solar radiation variables that are more likely to be uniform within the small area studied.

Given we did not have individual exposure measurements on which to form hypotheses, this study was necessarily exploratory. Given the exploratory nature there was multiple testing, however this was unavoidable given we had no literature on which to base our hypotheses for this outcome. During the outbreak there was a small increase in non-cardiac chest pain, however this may represent background variation and we do not believe it has any large impact on our results, if anything the inclusion of non-cardiac chest pain if not related to weather and pollution would bias our results towards the null. Future individual-level study of this phenomenon, using medical record reviews or patient interviews, could provide greater certainty into the direct cause of such 'outbreaks'. Nevertheless, this study does highlight several potential new areas of environmental health outcomes research, particularly the role of environmental factors in patient acuity, the mechanism of solar radiation's affect on health outcomes, small area variation in health outcomes and the relative significance of minimum daily PM_10 _measures compared with other measures of PM_10 _exposure.

Given the complexity of the modelling process used in assessing an environmental cause, future work could be concentrated on establishing mechanisms that would enable more real-time access to environmental data and timely, automated and synchronized analysis of environmental and health outcome data to be built into syndromic surveillance systems. With real-time access to multiple environmental variables, real-time modelling could be conducted to provide an assessment of environmental involvement as part of the same process that signals an increase. This factor is particularly important given the original focus of syndromic surveillance is on detecting bioterrorism. Improving our ability to exclude naturally occurring causes of disease could allow greater confidence in suspecting nefarious human intent behind disease outbreaks.

## Conclusion

We found that minimum daily PM_10 _levels have an important association with daily counts of emergency department visits for imminently or immediately life threatening cardiovascular disease or chest pain syndromes, and that this may reflect prolonged exposure during the day. However, the factors that best explained the excess attendances with this outcome in April-May 2005 were likely to be of photochemical origin, given the observed associations with ozone, solar radiation and temperature during that period.

This is the first reported study to demonstrate the value of syndromic surveillance for identifying short-term health problems that could have arisen from environmental factors. It has also raised a number of new hypotheses that could be explored in future air pollution and environmental health research.

## Abbreviations

AIC : Akaike information criteria;

CBMS : Community based monitoring station;

CI : Confidence interval;

CO : Carbon Monoxide;

ICD : International Classification of Diseases;

NEPH : Particulate matter as determined by integrating nephelometry;

NO : Nitrous oxide;

NO_2 : _Nitrous dioxide;

NSW : New South Wales;

PM_10 : _Particulate matter less than 10 microns;

PM_2.5 : _Particulate matter less than 2.5 microns;

RR : rate ratio.

## Competing interests

The author(s) declare that they have no competing interests.

## Authors' contributions

RMT participated in the study design, statistical analysis and drafted the methods and results in the manuscript. DJM conducted the literature review, participated in the study design and drafted the discussion and conclusions. WZ participated in the study design, statistical analysis and drafted the introduction. AW participated in the study design, mapped the study area and edited the manuscript. GA conducted the analysis of the patient characteristics and edited the manuscript. All authors read and approved the final manuscript.
